# Inhibition of mTORC1 Signaling Reverts Cognitive and Affective Deficits in a Mouse Model of Parkinson’s Disease

**DOI:** 10.3389/fneur.2018.00208

**Published:** 2018-04-09

**Authors:** Débora Masini, Alessandra Bonito-Oliva, Maëlle Bertho, Gilberto Fisone

**Affiliations:** Department of Neuroscience, Karolinska Institutet, Stockholm, Sweden

**Keywords:** Parkinson’s disease, mammalian target of rapamycin, rapamycin, ribosomal protein S6 kinase, PF-4708671, depression, anxiety, cognition

## Abstract

Non-motor symptoms, including cognitive deficits and affective disorders, are frequently diagnosed in Parkinson’s disease (PD) patients and are only partially alleviated by dopamine replacement therapy. Here, we used a 6-hydroxydopamine (6-OHDA) mouse model of PD to examine the effects exerted on non-motor symptoms by inhibition of the mammalian target of rapamycin complex 1 (mTORC1), which is involved in the control of protein synthesis, cell growth, and metabolism. We show that rapamycin, which acts as an allosteric inhibitor of mTORC1, counteracts the impairment of novel object recognition. A similar effect is produced by PF-4708671, an inhibitor of the downstream target of mTORC1, ribosomal protein S6 kinase (S6K). Rapamycin is also able to reduce depression-like behavior in PD mice, as indicated by decreased immobility in the forced swim test. Moreover, rapamycin exerts anxiolytic effects, thereby reducing thigmotaxis in the open field and increasing exploration of the open arm in the elevated plus maze. In contrast to rapamycin, administration of PF-4708671 to PD mice does not counteract depression- and anxiety-like behaviors. Altogether, these results identify mTORC1 as a target for the development of drugs that, in combination with standard antiparkinsonian agents, may widen the efficacy of current therapies for the cognitive and affective symptoms of PD.

## Introduction

Cognitive impairment and affective disorders are frequently diagnosed in patients with Parkinson’s disease (PD) and represent a major clinical challenge, in addition to the classic motor symptoms (1–5). Dementia develops in about 40% of PD patients and is often preceded by mild cognitive impairments, which compromise attentional, executive, and visuospatial functions. These latter ailments often develop before the onset of cardinal motor symptoms and are present in about 20% of PD patients at the time of diagnosis (4, 5). A significant proportion of PD patients are also affected by anxiety and depression, which appear in the early stages of the disease and are often refractory to dopamine replacement therapies (1, 3). Non-motor symptoms represent a serious challenge to the quality of life for both patients and their families, prompting the search for more effective therapies.

The mammalian target of rapamycin (mTOR) signaling pathway is involved in multiple aspects of cognitive processes. mTOR is the key catalytic component of two large multimeric complexes: mTOR complex 1 (mTORC1) and 2 (mTORC2) (6, 7). mTORC1 regulates a variety of cellular functions, including cell growth and proliferation, autophagy and protein synthesis, whereas mTORC2 participates in the control of cytoskeletal dynamics and cell size.

Two of the main downstream targets of mTORC1, the ribosomal protein S6 kinase (S6K) and the eukaryotic initiation factor 4E-binding protein (4E-BP), promote mRNA translation *via* activation of downstream initiation and elongation factors (8–10). Activation of these signaling components modulates synaptic plasticity and affects cognition through spatial and temporal coordination of protein synthesis. Thus, mTORC1 signaling is required for long-term potentiation in the hippocampus, and for memory formation and consolidation (11, 12).

Excessive activation of mTORC1 is linked to intellectual disabilities, including tuberous sclerosis (13, 14), fragile X syndrome [(15) but see also (16)] and Down syndrome (17). Notably, the cognitive impairment observed in animal models of tuberous sclerosis and Down syndrome is counteracted by rapamycin, a selective inhibitor of mTORC1 (13, 14, 18).

Dysregulated mTOR transmission is also implicated in affective disorders. The current prevailing hypothesis is that decreased mTORC1 activity in different cortical regions is associated with depression whereas augmented mTORC1 activity, such as that produced by the NMDA receptor agonist ketamine, reverts these conditions (19–22). However, studies in animal models have shown that subchronic administration of rapamycin reduces depressive-like behaviors (23), prompting further analysis of the actions of this drug on emotional deficits.

In this study, we used a mouse model to examine the effects of rapamycin and PF-4708671, a selective S6K inhibitor (24), to counteract memory impairment, depressive- and anxiety-like behaviors associated with PD. Our results indicate that inhibition of mTORC1 with rapamycin may represent a potential approach to the combined treatment of these disorders.

## Materials and Methods

### Animals

Male C57BL/6J mice (3 months old; 25–30 g; Jackson Laboratory, ME, USA) were housed under a 12 light-dark cycle with food and water *ad libitum*. All experiments were carried out in accordance with the guidelines of Research Ethics Committee of Karolinska Institutet and Swedish Animal Welfare Agency. All efforts were made to minimize animal suffering and to reduce the number of animals used.

### Drugs

6-hydroxydopamine hydrochloride (6-OHDA; Sigma-Aldrich, Stockholm, Sweden) was dissolved in 0.02% ascorbic acid in saline at a concentration of 4 µg/µL and injected directly into the dorsal striatum. Rapamycin (LC Laboratories, Woburn, MA, USA) was dissolved in 5% dimethyl sulfoxide (DMSO), 5% Tween20, 15% polyethylene glycol (PEG), and distilled water to a final concentration of 5 mg/kg, and administered intraperitoneally (i.p.) in a volume of 2 mL/kg for three consecutive days, and then 30 min preceding the open field (OF), the elevated plus maze (EPM), and the forced swim test (FST). Rapamycin was also injected 30 min prior to all phases of the novel object recognition (NOR) test. PF-4708671 (MedChem Express, Monmouth Junction, USA) was dissolved in 17% DMSO, 10% Tween80, in saline and injected (50 mg/kg in 5 mL/kg volume, i.p.) 1 h prior to each experiment (OF, EPM, FST, familiarization and test phases of NOR).

### 6-OHDA Lesion

Mice were anesthetized with 4% isofluorane and positioned in a stereotaxic frame (David Kopf Instruments, Tujunga, CA, USA) equipped with a heating pad to maintain normothermia. All animals were injected subcutaneously with 0.1 mg/kg of Temgesic before surgery. Partial dopamine depletion was induced by injecting each striatum with 1 µL of 6-OHDA according to the following coordinates (mm): anteroposterior + 0.6; mediolateral ± 2.2; and dorsoventral −3.2 (25). Control mice received a sham lesion, consisting of bilateral injections (1 µL of vehicle). After surgery, the animals were allowed to recover for 3 weeks.

### Behavioral Tests

Each mouse was subjected to sequential tests performed according to their increasing averseness (i.e., OF, NOR, EPM, and FST). Each test was separated by 4–7 days, during which animals were left undisturbed.

#### Open Field (OF) and Thigmotaxis

In this test, the preferential exploration of the peripheral zone of the OF, referred to as thigmotaxis, is considered an index of anxiety (26–28). Thigmotaxis was evaluated in a box (38 cm × 38 cm × 28 cm) divided into peripheral and central zones (defined as body center beyond 10 cm from wall). Each mouse was allowed to explore the apparatus for 15 min and its behavior was recorded by a video camera connected to an automated tracking system (Ethovision XT-10, Noldus, The Netherlands). The percentage of time spent by the animals exploring the center zone of the apparatus was measured and represented as a time course scatter plot (30 s time sampling intervals, one-zero sampling method). Curve fitting with nonlinear regression was used to generate trend lines. The cumulative time in the center zone was calculated as percentage of the total experimental time.

#### Novel Object Recognition (NOR)

The NOR test is based on the natural preference of rodents for novel objects and is commonly employed to assess memory function (27, 28). Mice were first habituated for 3 days (20 min/day) to the experimental cage (38 cm × 38 cm plastic chamber). On the familiarization phase (day 4), two identical objects (white plastic cylinders 3 cm high and 1 cm radius) were placed in the back left and right corners of the cage, 10 cm from the walls. Mice were placed near the wall opposite to the objects and allowed to explore for 15 min. During the test (day 5), one of the two (familiar) objects was replaced with a novel object (plastic orange object of comparable size). Mice were placed in the apparatus and left free to explore for 5 min. The experiment was video-recorded and object exploration (time during which the mouse nose was in contact with the object or directed toward it at a distance ≤ 2 cm) was measured by an observer blind to groups and treatments. Two measures were considered: (1) the total exploration time (s) spent by the animal interacting with the two objects during the test and (2) the exploration time (%) spent by the animal interacting with the novel object over the total exploration time (e.g., [novel/(familiar + novel)] × 100) during the test. Example tracings shown in the NOR test were generated by plotting the *x*,*y* positions of the nose-point, detected every 0.2 s and color coded according to the behavior being assessed.

#### Elevated Plus Maze (EPM)

The EPM test is commonly used to evaluate anxiety-like behavior in mice. The test is based on the natural preference of rodents for closed spaces, and the propensity to avoid the open arms is considered an index of anxiety (29). The apparatus is composed of four gray plastic arms, arranged as a cross and located 40 cm above the plane of a laboratory bench. Two arms, opposite to each other, are enclosed by lateral walls (70 cm × 6 cm × 40 cm) and the other arms are without walls (70 cm × 6 cm × 0.75 cm); the closed and open arms delimit a small square area (6 cm × 6 cm) named center. Each mouse was placed in the center of the maze facing one of the open arms away from the experimenter, and its behavior was video-recorded for 5 min. The time spent by the mice in each of the three compartments (open, close, center zones) and the distance covered were measured with Ethovision XT-10. Head dips, defined as events in which the mouse nose-point was beyond the borders of the maze, were scored. Position heat maps were generated by averaging the proportion of track found in each location per animal. Range of colors was calculated by comparing the location frequencies of all subjects, with images representing the group averages.

#### Forced Swim Test (FST)

The FST is a standard paradigm to evaluate depression-like behavior in rodents (30). In this test, each mouse was placed in a glass cylinder (25 cm in height and 17 cm in diameter), filled up to 16 cm with water at a temperature of 23°C and let swim for 10 min. At the end of the test, the mouse was removed from the cylinder, gently dried, and placed in a new cage on a warm pad for at least 20 min. The test was video-recorded and analyzed both manually, by an observer blind to groups, and automatically (Ethovision XT-10). The immobility time (defined as the time spent by the mouse floating, with only minimal movements to keep the head above the water surface) was measured and considered as an index of depression. Climbing was defined by forceful thrash movements of the forelimbs against the walls of the cylinder and concomitant displacement of body center <1.6 cm below water surface (calibrated to animal body size).

### Statistical Analyses

Behavioral data were analyzed with one-way ANOVA (multiple groups) or two-way repeated measures ANOVA (multiple groups with two measures per subject), and *post hoc* comparisons between groups were made with Fisher’s LSD test or Holm–Sidak’s test, respectively. Data that did not comply with normality assumptions (Brown–Forsythe test for SD) were analyzed with Kruskal–Wallis test, followed by Dunn’s multiple comparison test. *p* ≤ 0.05 were considered significant and all data are presented as mean ± SEM.

## Results

Previous work showed that mice with a partial 6-OHDA lesion of the dopaminergic system display memory deficit and affective disturbances reminiscent of early stage PD (31–33). In the first series of experiments, this model was employed to examine the effects of rapamycin and PF-4708671 on the disruption of long-term memory. Four groups of animals were used: sham-lesion (Sham) mice treated with vehicle, 6-OHDA lesion (Lesion) mice treated with vehicle, Lesion mice treated with rapamycin (Lesion Rapa), and Lesion mice treated with PF-4708671 (Lesion PF).

**Figure 1:**
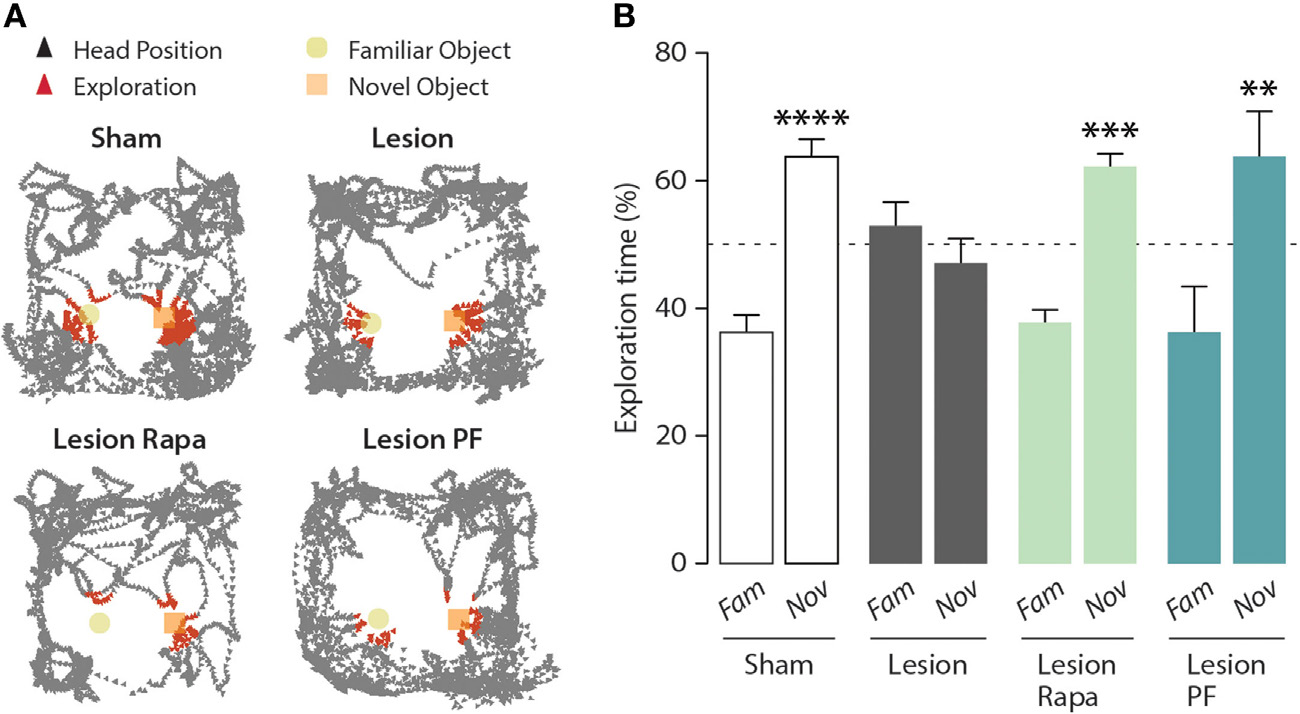
Inhibition of mammalian target of rapamycin complex 1, or its downstream target S6 kinase, rescues novel object recognition (NOR) memory in a mouse model of Parkinson’s disease. NOR test performance in control (Sham) mice treated with vehicle (*n* = 16), and Lesion mice treated with vehicle (*n* = 12), rapamycin (Lesion Rapa, *n* = 16), or PF-4708671 (Lesion PF, *n* = 8). **(A)** Representative traces of mice performing the NOR test. Triangles indicate the head position during the test (in black) and during exploratory behavior (in red). **(B)** Time spent exploring the familiar (Fam) or novel (Nov) object over a 5 min test. Data are expressed as percent of total exploration time and represented as mean ± SEM. **** *p* = 0.0001, *** *p* = 0.0003, and ** *p* = 0.003 vs. Fam within groups (two-way ANOVA followed by Fisher’s *post hoc* comparison).

As shown in Figure 1, the 6-OHDA lesion abolished the ability of the mice to distinguish between a familiar and a novel object. Subchronic administration of rapamycin, starting 4 days preceding the test, reverted the impairment of NOR produced by partial dopamine depletion.

mTORC1 regulates two major downstream effector targets involved in the modulation of protein synthesis: 4E-BP and S6K (6, 7). In order to determine the contribution of these two signaling components, we compared the effect of rapamycin, which prevents mTORC1-mediated regulation of both 4E-BP and S6K, with that of the selective S6K inhibitor PF-4708671 (24). Similar to rapamycin, PF-4708671 reverted the impairment of NOR observed in the Lesion group. Two-way ANOVA indicated significant group × object interaction (*F*_3,48_ = 5.10, *p* = 0.004), Fisher’s *post hoc* comparison (Figure 1B).

It should be noted that, under these experimental conditions, Lesion mice treated with PF-4708671 showed a 60% reduction in the overall object exploration, as compared with the other groups. However, this reduction was not accompanied by reduced motor activity and, importantly, did not affect their ability to perform the task (data not shown).

**Figure 2:**
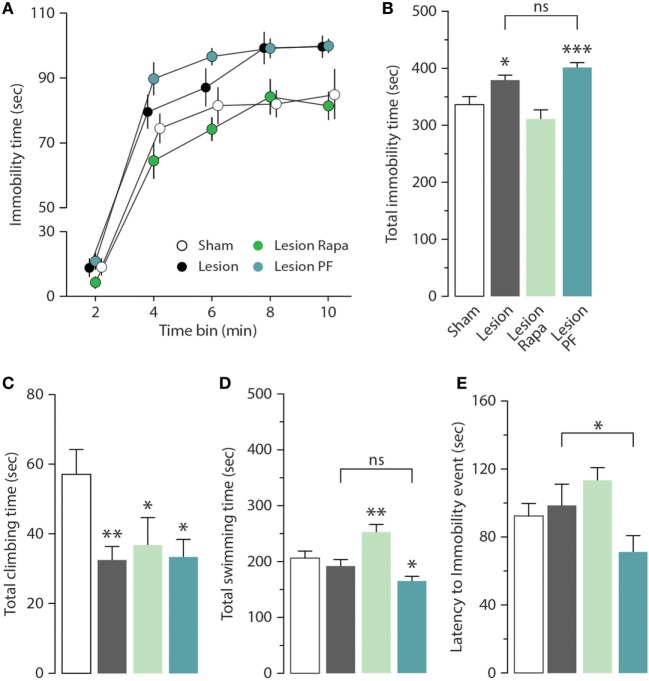
Rapamycin reverts depression-like behavior in a mouse model of Parkinson’s disease. Immobility time in the forced swim test (FST) was measured in Sham (*n* = 12), and Lesion mice treated with vehicle (*n* = 12), rapamycin (Lesion Rapa, *n* = 12), or PF-4708671 (Lesion PF, *n* = 12). **(A)** Time course (2 min bins) over the 10 min FST test. Repeated measures two-way ANOVA followed by Holm–Sidak’s *post hoc* indicated a significant difference *p* = 0.02 for Lesion vs. Sham and *p* = 0.0006 for Lesion PF vs. Sham. **(B)** Total immobility time (s) during the 10-min FST test. * *p* = 0.01 and *** *p* = 0.0002 vs. Sham (one-way ANOVA followed by Fisher’s *post hoc* comparison). **(C)** Total time (s) spent climbing. * *p* = 0.03 and 0.01, ** *p* = 0.007 vs. Sham (one-way ANOVA followed by Fisher’s *post hoc* test). **(D)** Total time (s) spent swimming. * *p* = 0.01, ** *p* = 0.006 vs. Sham (one-way ANOVA followed by Fisher’s *post hoc* comparison). **(E)** Latency (s) to first immobility event. * *p* = 0.04 Lesion vs. Lesion PF (one-way ANOVA followed by Fisher’s *post hoc* comparison). Data are presented as mean ± SEM. Groups and treatments are as indicated in (B).

We next examined the effect of rapamycin and PF-4708671 on the depression-like behavior produced by partial dopamine depletion. As previously reported (31), Lesion mice displayed increased immobility in the FST (Figures 2A,B). Time course analysis (2 min bins) indicated that the highest immobility time of Lesion mice occurred in the second half of the test (significant group × time interaction two-way repeated measures ANOVA, *F*_3,44_ = 13.02, *p* < 0.0001, followed by Holm–Sidak’s *post hoc* comparison: bin 8 *p* = 0.007, bin 10 *p* = 0.02) (Figure 2A). Cumulative analysis showed that the increase in immobility time produced by the 6-OHDA lesion was reverted by rapamycin, but not by PF-4708671 (one-way ANOVA, *F*_3,44_ = 13.01, *p* < 0.0001, followed by Fisher’s *post hoc* comparison) (Figure 2B).

The depression-like response in the FST was further analyzed by measuring climbing (Figure 2C), swimming time (Figure 2D), and latency to the first immobility event (Figure 2E). We found that climbing activity was reduced in 6-OHDA lesion mice (one-way ANOVA, *F*_3,44_ = 3.29, *p* = 0.03, followed by Fisher’s *post hoc* comparison), with no effect on swimming time (one-way ANOVA, *F*_3,44_ = 10.52, *p* < 0.0001, followed by Fisher’s *post hoc* comparison), or latency to immobility (one-way ANOVA, *F*_3,44_ = 3.43, *p* = 0.02 followed by Fisher’s *post hoc* comparison). Treatment with rapamycin increased swimming time (Figure 2D) without affecting climbing activity (Figure 2C) or latency to immobility (Figure 2E). PF-4708671 did not modify the performance of 6-OHDA lesion mice with regard to climbing and swimming (Figures 2C,D), and reduced latency to immobility (Figure 2E).

**Figure 3:**
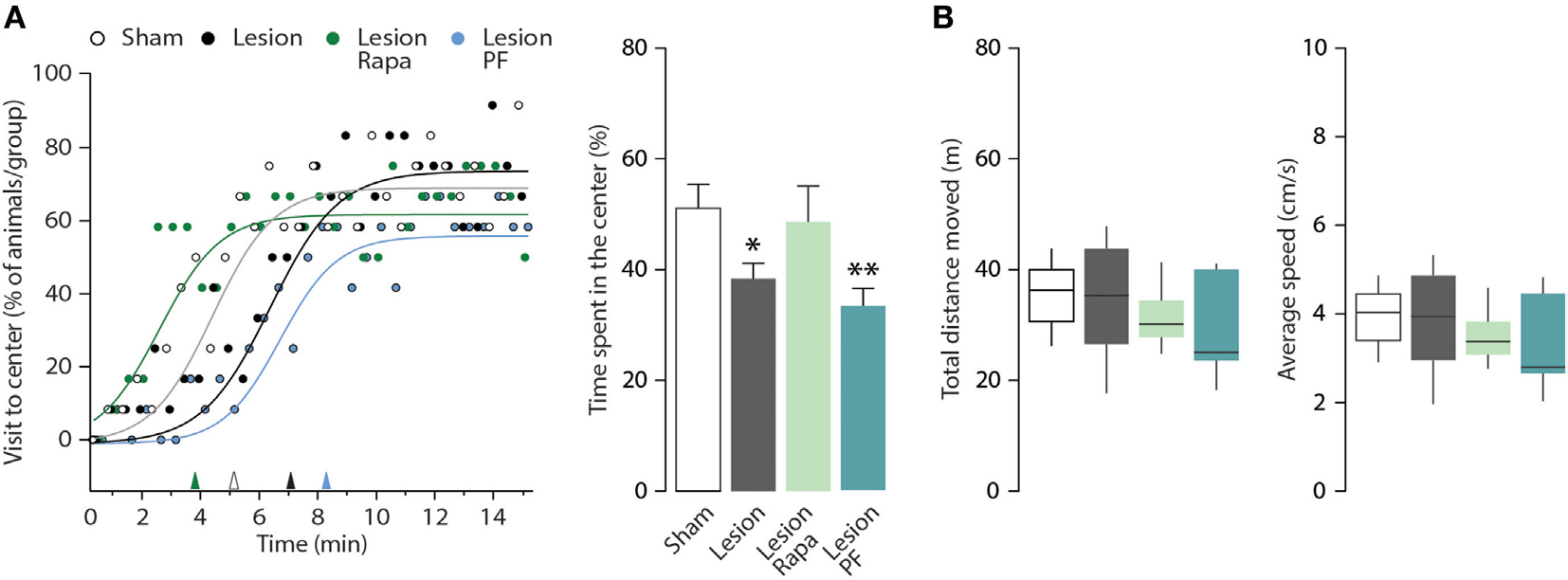
Rapamycin counteracts thigmotaxis in a mouse model of Parkinson’s disease. Open field (OF) performance of Sham (*n* = 12), and Lesion mice treated with vehicle (*n* = 12), rapamycin (Lesion Rapa, *n* = 12), or PF-4708671 (Lesion PF, *n* = 12) during the 15-min test. **(A)** Left panel shows the percentage of animals that visited the center of the OF, expressed as time course. The performance is graphed as 30-s time points (circles) and best-fitting curves. Triangles on the *x*-axis indicate the latency (min) for 50% of the animals in each group to explore the center zone of the OF. Right panel shows the time spent in the center zone, expressed as percent over the total duration of the test. Data are presented as mean ± SEM. * *p* = 0.05, ** *p* = 0.007 vs. Sham (one-way ANOVA followed by Fisher’s *post hoc* comparison). **(B)** Tukey whiskers plots with the median, 10th, 25th, 70th, and 90th percentiles showing total distance moved in the OF (m) and average speed (cm/s). One-way ANOVA showed no differences between groups. Groups and treatments are as indicated in (A).

The mouse model of PD utilized in this study displays anxiety-like behavior in multiple paradigms (31, 33). In this study, we used the OF test to evaluate thigmotaxis as an index of anxiety in Sham and Lesion mice treated with vehicle, rapamycin or PF-4708671. The number of visits to the center zone of the OF (measured as visits/30 s, during a period of 15 min) is increased in all groups over time (two-way repeated measures ANOVA indicates a significant effect of time F_28,1232_ = 16.14, *p* < 0.0001) (Figure 3A, left panel). We also observed a significant effect of treatment during the course of the experiment (two-way repeated measures ANOVA, group × time interaction, *F*_84,1232_ = 1.41, *p* = 0.0098). Best-fitting curves showed that Sham mice began exploring the center zone of the OF earlier than Lesion mice (Figure 3A, left panel). In line with this measurement, thigmotaxis was increased in Lesion mice, as indicated by reduced time spent in the center zone (one-way ANOVA, *F*_3,44_ = 3.59, *p* = 0.02 followed by Fisher’s *post hoc* comparison) (Figure 3A, right panel). The increase in thigmotaxis observed in Lesion mice was reverted by rapamycin, but not by PF-4708671. Neither lesion nor drug treatments affected the distance (m) covered by the animals or their average speed (cm/s) (Figure 3B).

**Figure 4:**
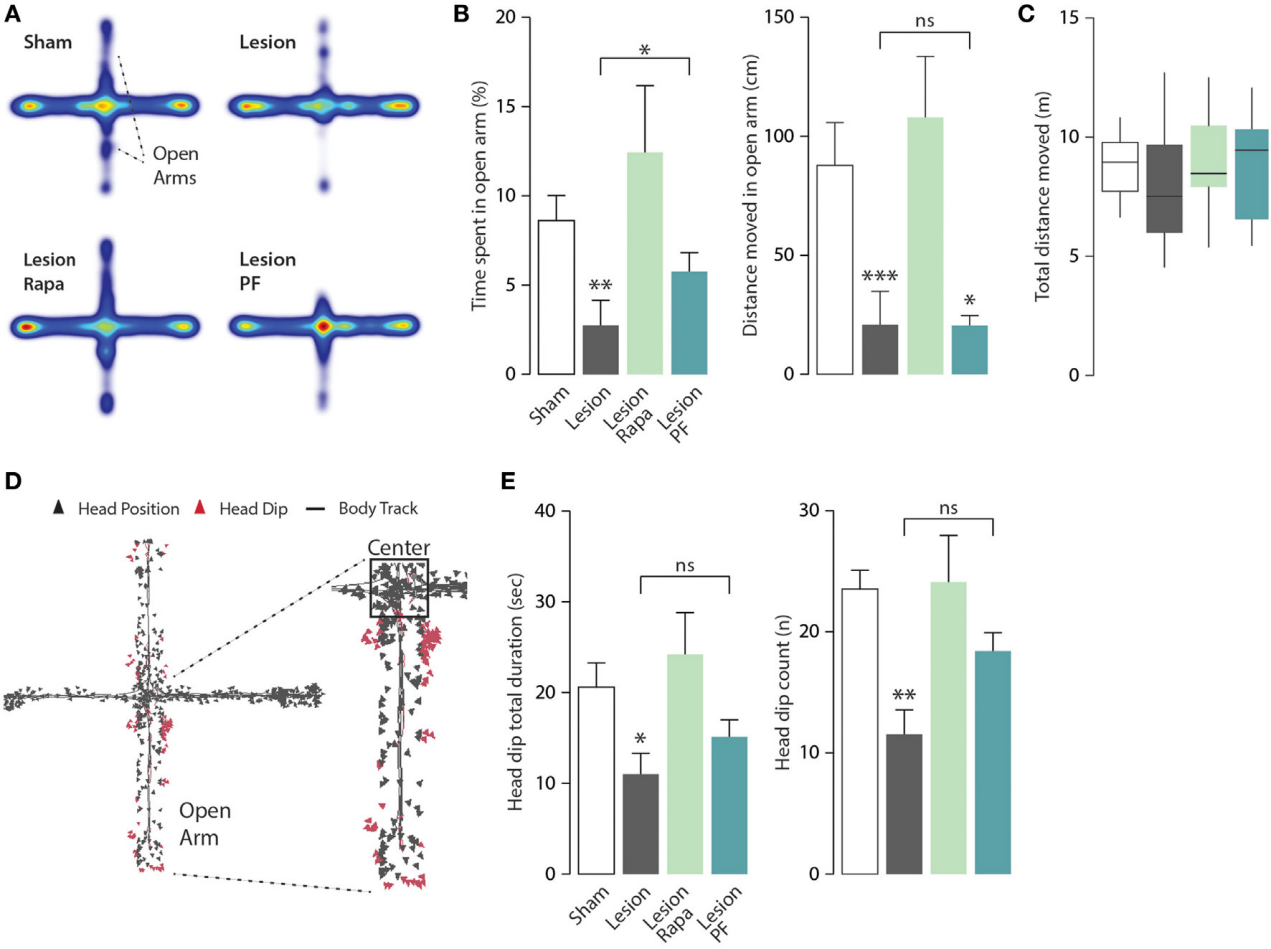
Rapamycin abolishes anxiety-like behavior in a mouse model of Parkinson’s disease. Elevated plus maze (EPM) test performance in Sham mice treated with vehicle (*n* = 12), and Lesion mice treated with vehicle (*n* = 12), rapamycin (Lesion Rapa, *n* = 12), or PF-4708671 (Lesion PF, *n* = 12). **(A)** Group average heat map locations where range of colors was calculated comparing location frequencies over all subjects. **(B)** Time spent in the EPM open arm expressed as percent of total time (left panel) and total distance moved (cm) (right panel) during the 5-min test. ** *p* = 0.005, * *p* = 0.03 vs. Sham for time, and *** *p* = 0.0005, * *p* = 0.048 vs. Sham for distance (Kruskal–Wallis test followed by Dunn’s *post hoc* comparison). **(C)** Tukey whiskers plots with the median, 10th, 25th, 70th, and 90th percentiles showing total distance (m) covered in the EPM. One-way ANOVA showed no differences between groups. Groups and treatments are as indicated in (B). **(D)** Head dip analysis carried out by tracking nose-point position, as an additional marker of anxiety. Triangles indicate the head position during the test with head dip events in red. **(E)** Total duration (s) of head dips (left panel) and number of events (right panel). Data are presented as mean ± SEM. * *p* = 0.03 and ** *p* = 0.002 vs. Sham group (one-way ANOVA followed by Fisher’s *post hoc* comparison).

Mice were further tested for anxiety-like behavior in the EPM apparatus. Heat maps with group average were generated to allow visualization of exploration patterns in response to the different treatments (Figure 4A). In line with previous work, Lesion mice spent significantly less time in the open arms of the EPM compared with Sham mice (31, 33). We observed that this effect was reversed when Lesion mice were treated with rapamycin (Kruskal–Wallis, *p* = 0.006, followed by Dunn’s *post hoc* comparison). A partial reduction of the effect of the 6-OHDA lesion was also observed in response to PF-4708671 (Dunn’s *post hoc* comparison, Lesion vs. Lesion PF, *p* = 0.03) (Figure 4B, left panel).

The similarity of the heat maps generated from Lesion and Lesion PF mice prompted a further analysis of the activity of each experimental group in the open arm of the EPM, to better understand the effects of rapamycin and PF-4708671. Analysis of the distance moved (cm) in the open arms showed no difference in motor activity, which should be regarded as a marker of exploratory behavior, between Lesion, and Lesion PF mice (Kruskal–Wallis, *p* < 0.0001, followed by Dunn’s *post hoc* comparison) (Figure 4B, right panel). Notably, the reduced exploration of the open arm observed in these mice cannot be explained by a decrease in overall mobility since this parameter was comparable in all groups, during the 5-min test (Figure 4C). In line with these observations, Lesion PF mice spent more time in the center of the apparatus in comparison to the other groups (Kruskal–Wallis, *p* = 0.01, followed by Dunn’s *post hoc*) (Figure 4A, cf. red color).

In the EPM test, head dip events are regarded as an additional indication of reduced anxiety (34). Therefore, we measured this behavior with automated tracking of nose-point position (Figure 4D). The total duration of head dip events (s) and their number were reduced in Lesion mice and these effects were reverted by rapamycin, but not by PF-4708671 (for head dip duration: one-way ANOVA, *F*_3,44_ = 3.63, *p* = 0.02 followed by Fisher’s *post hoc* comparison; for head dip number: one-way ANOVA, *F*_3,44_ = 5.51, *p* = 0.002, followed by Fisher’s *post hoc* comparison) (Figure 4E). No effect was found in the average duration of individual head dip events. Altogether, the analyses of anxiety-like behaviors indicate that rapamycin, but not PF-4708671, abolishes anxiety-like behaviors in experimental parkinsonism.

## Discussion

This study shows that inhibition of mTORC1, or its downstream target S6K, counteracts the memory deficit observed in a mouse model of early stage PD. It also shows that depression- and anxiety-like behaviors are eliminated by mTORC1 inhibition, but not by selective blockade of the mTORC1 downstream target, S6K.

The mouse model used in this study is based on a partial bilateral lesion with 6-OHDA, leading to 65–75% loss of dopaminergic nigrostriatal innervation and striatal dopamine levels (31, 32). These reductions reproduce an early stage of PD, characterized by mild changes in gate dynamics (31), which are unlikely to interfere with the assessment of cognitive and affective parameters.

We found that subchronic administration of rapamycin, which effectively reduces mTORC1 activity in the brain (35), abolishes the impairment of long-term NOR produced by a partial lesion of the dopamine system. Rapamycin acts by preventing the phosphorylation of S6K and 4E-BP, which in turn regulate two parallel signaling branches implicated in the control of protein synthesis, and in multiple aspects of synaptic plasticity and memory (11, 12). Our results indicate that selective inhibition of S6K with PF-4708671 is sufficient to rescue memory performance. Interestingly, PF-4708671 has also been shown to rescue hippocampal long-term potentiation and counteract behavioral abnormalities in mouse models of Angelman and fragile X syndromes (36, 37).

Rapamycin and PF-4708671 have been previously reported to re-establish cognitive performance in pathological models characterized by abnormal mTORC1 signaling and protein translation (38, 39). Such dysregulation has not been demonstrated in the model of PD utilized in this study; thus, mTORC1, or S6K inhibition, is likely to correct memory deficits independently of a preexisting condition of mTORC1 hyperactivation. In line with this possibility, a clinical study showed that administration of the rapamycin analog everolimus, following cardiac transplant, a condition which is not associated with abnormal mTORC1 regulation, results in a significant improvement of memory and affective performance (40). Interestingly, this effect was proposed to occur, at least in part, through reduction of brain inflammation, which is commonly associated with neurodegenerative disorders including PD (41).

In addition to cognitive impairment, the mouse model utilized in this study reproduces affective symptoms typically observed in PD patients, such as depression and anxiety. We found that rapamycin counteracts the depression-like behavior manifested by PD mice as increased immobility in the FST. Notably, we observed that the anti-depressant effect of rapamycin is exerted by promoting swimming, but not climbing, which is regarded as a behavioral component related to motor stimulation rather than anti-depressant properties (42).

The finding that rapamycin reduces depression-like behavior contrasts with previous studies indicating that reduced mTOR signaling is associated with depression (19–22). In this regard, our results are more in line with the observation that subchronic administration of rapamycin, albeit at higher doses than those used in the present study, exerts anti-depressant effects in the FST and tail suspension tests (23).

In contrast to the results obtained with rapamycin, we did not observe any decrease in immobility time in the FST when PD mice were treated with PF-4708671. This suggests that the anti-depressant action of rapamycin depends on concomitant inhibition of the 4E-BP and S6K signaling cascades or that additional alternative mechanisms downstream of mTORC1 are required. For instance, rapamycin may reduce depression by promoting autophagy, which is negatively regulated by mTORC1 through inhibition of the mammalian autophagy-initiating kinase Ulk1 (43). In support of this possibility, several agents exerting anti-depressant actions, including lithium, citalopram, and trehalose, have been shown to induce autophagy (44–46).

Rapamycin counteracts the anxiety-like behavior observed in the mouse model of PD. In particular, this drug normalizes the time spent by PD mice in the center zone of the OF, thereby reducing thigmotaxis. A similar normalization was also observed in the EPM test, in which rapamycin increased the propensity of PD mice to explore the open arm of the apparatus. Selective inhibition of S6K with PF-4708671 did not produce a reduction of anxiety-like behaviors comparable to that observed with rapamycin. Thus, PF-4708671 did not reduce thigmotaxis in the OF and only partially reverted anxiety-like behavior in the EPM test. Although PF-4708671 increased the time spent by PD mice in the exposed area of the apparatus, it failed to induce a full exploration of the open arm. Moreover, and in contrast with rapamycin, administration of PF-4708671 did not counteract the reduction in head dip behavior, which is regarded as another indicator of anxiety. Altogether, these results indicate that rapamycin is capable of fully rescuing affective behavior in a mouse model of PD, and that this effect likely requires blockade of multiple downstream targets of mTORC1.

In conclusion, we show that inhibition of mTORC1 with rapamycin effectively counteracts memory deficit and mood disorders in a model of PD. We also show that inhibition of S6K, a well-characterized target of mTORC1, partially reproduces these effects by rescuing memory performance. Further studies will be necessary to fully characterize the action of rapamycin and identify additional components of the mTORC1 signaling machinery that may represent additional targets for the treatment of psychiatric symptoms associated with PD.

## Ethics Statement

All experiments were carried out in accordance with the guidelines of Research Ethics Committee of Karolinska Institutet and Swedish Animal Welfare Agency.

## Author Contributions

GF conceived the project. DM and ABO designed experiments with contributions from all authors. DM and MB performed experiments and statistical analysis. GF and DM wrote the manuscript with contributions from all authors. GF supervised all aspects of the work.

## Conflict of Interest Statement

The authors declare that the research was conducted in the absence of any commercial or financial relationships that could be construed as a potential conflict of interest.
